# Pallidal Versus Subthalamic Deep-Brain Stimulation for Generalized Isolated Dystonia: A Retrospective Study

**DOI:** 10.3390/jcm13164902

**Published:** 2024-08-20

**Authors:** Jingchao Wu, Guanyu Zhu, Yifei Gan, Fangang Meng, Anchao Yang, Jianguo Zhang

**Affiliations:** 1 Department of Neurosurgery, Beijing Tiantan Hospital, Capital Medical University, Beijing 100070, China; 2 Department of Neurosurgery, Tianjin Huanhu Hospital, Tianjin 300000, China

**Keywords:** DBS, STN, GPi, dystonia

## Abstract

**Objectives:** Deep-brain stimulation (DBS) has been used for the treatment of medically refractory dystonia with excellent results. In this study, we compared in detail the therapeutic advantages of two DBS targets for generalized isolated dystonia. **Methods:** In this retrospective study, we recruited 29 patients with generalized isolated dystonia who had undergone DBS treatment targeting either the globus pallidus interna (GPi) or the subthalamic nucleus (STN) in the Department of Functional Neurosurgery at Tiantan Hospital, Beijing, China, between January 2016 and December 2021. The movement and disability subscales of the Burke–Fahn–Marsden dystonia rating scale (BFMDRS) were used to assess the severity of their dystonic symptoms and their activities of daily living, respectively. SF-36 was used to evaluate the patients’ health-related quality of life. **Results:** The percentage improvement in the BFMDRS-M score at 6 months relative to the baseline score was clearly higher in the STN group (63.91%) than in the GPi group (38.36%). At the 3-, 6-, and 12-month follow-ups, the percentage improvement in arm symptoms was significantly higher after DBS of the STN (70.64%, 80.66%, and 76.89%, respectively) than after stimulation of the GPi (36.75%, 34.21%, and 38.47%, respectively). At 12 months after surgery, patient quality of life had improved on all SF-36 subscales in both groups. **Conclusions:** STN-DBS may have more advantages than GPi-DBS in patients with obvious arm dystonia. STN-DBS had a better clinical effect than GPi-DBS within 6 months after surgery.

## 1. Introduction

Dystonia is defined as a group of movement disorders characterized by sustained or intermittent muscle contractions that cause twisting, repetitive movements, or abnormal posture. Dystonia can be classified based on clinical characteristics such as age when symptoms start, the way it affects different parts of the body, the pattern of occurrence over time, and any accompanying movement disorders or neurological features. Additionally, it can also be classified based on the underlying causes, which include nervous system pathology and genetic inheritance [1]. In isolated dystonia, dystonia and/or tremor are the only symptoms and signs, and no other neurological abnormalities are present [2]. Generalized dystonia must involve the trunk and at least two other sites [1]. The medical treatment of dystonia can be difficult, with intolerable adverse effects or unsustained symptom relief. The effects of injections of botulinum toxin are always transient, and this treatment is appropriate only for specific types of dystonia, such as focal dystonia. It is unsuitable for patients with generalized dystonia because the affected muscles are widely distributed [3].

In recent years, deep-brain stimulation (DBS) has been used for the treatment of refractory dystonia with excellent results. Although the globus pallidus interna (GPi) has been most frequently targeted with DBS in the treatment of isolated dystonia, many studies have demonstrated the safety and effectiveness of targeting the subthalamic nucleus (STN) with DBS [4,5,6]. To date, only one study [7] has compared the clinical outcomes of stimulating the two targets in patients with isolated dystonia. The study by Lin et al. [7] suggested that stimulating the STN produced a faster clinical reaction, whereas GPi stimulation was more effective in patients with axial symptoms. However, that retrospective study included patients with focal, segmental, multifocal, and generalized dystonia, and the high variability within each group may have affected the researchers’ ability to detect differences between the groups.

In this study, we compared the results of targeting either the GPi or STN in 29 patients with generalized isolated dystonia during the 12-month follow-up period after surgery. To the best of our knowledge, this is the largest retrospective study to compare in detail the therapeutic advantages of targeting GPi or STN in patients with generalized isolated dystonia.

## 2. Materials and Methods

### 2.1. Patients

In this retrospective study, we included patients with generalized isolated dystonia who had undergone DBS treatment targeting either the GPi or STN in the Department of Functional Neurosurgery at Tiantan Hospital, Beijing, China, between January 2016 and December 2021. All patients who met the criteria for DBS were clinically diagnosed with generalized, isolated, and medically intractable dystonia. Those patients who fulfilled the following criteria were included in this study: (1) normal preoperative brain magnetic resonance imaging (MRI) results and (2) no serious mental disorder, cognitive impairment, history of epilepsy, thalamotomy, or any other medical condition that made them unsuitable for surgery. Cases with missing follow-up and incomplete clinical data were excluded from the study population. Based on these criteria, we enrolled 29 patients with generalized isolated dystonia in this study. A total of 17 patients were treated with GPi-DBS, and 12 patients were treated with STN-DBS. The study was conducted in accordance with the principles of the Declaration of Helsinki and was approved by the Beijing Tiantan Hospital Ethics Committee (approval number: HX-A-2021006, approved on 6 April 2022). All patients or their families provided informed consent for the anonymous use of clinical data.

### 2.2. Treatment Procedure and Programming

Each patient underwent T1- and T2-phase 3.0 T MRI (MAGNETOM Prisma, Siemens, Erlangen, Germany) before DBS surgery. The Leksell-G stereotactic frame was installed on each patient’s head under local anesthesia, and a computed tomography (CT) scan was performed. The electrode implantation coordinates were confirmed after the target parameters on MRI, and the frame coordinates on CT were merged. Quadripolar DBS electrodes (PINS L302/L301, PINS Medical Co., Beijing, China; Medtronic 3387/3389, Medtronic Inc., Minneapolis, MN, USA) were implanted bilaterally into the GPi or STN with microelectrode recording under local anesthesia. The most favorable outcomes in the treatment of primary dystonia with DBS are observed when the electrodes are positioned in the posteroventral region of the GPi [8]. The GPi is positioned approximately 1–2 mm in front of the midpoint of the anteroposterior commissure with a lateral distance of 18–23 mm and a depth of 4–6 mm below the anteroposterior commissure. On the other hand, the STN is located around 2–3 mm behind the midpoint of the anteroposterior commissure with a lateral distance of 12–14 mm and a depth of 4–6 mm below the plane of the anteroposterior commissure. An implantable pulse generator (IPG; PINS G102 or G102RZ, Medtronic Activa RC) was placed subclavicularly under general anesthesia. A thin-slice brain CT scan was performed after the surgery, and the position of the lead was determined by merging the scan with the preoperative image.

The IPG was programmed 1 month after the operation. Each contact was activated and tested. We determined the optimal stimulation parameters based on the response of each patient. Lower stimulation parameters were set initially, and then they were gradually adjusted at outpatient consultations or with a telemedical application during follow-up. Our programming mainly followed the experience of West Toronto Hospital [9]. Specifically, for STN-DBS, we adjusted the voltage based on the experience at West Toronto Hospital.

### 2.3. Clinical Evaluation

Symptom assessment was based on videos recorded at baseline and at 1, 3, 6, and 12 months after the operation. The movement and disability subscales of the Burke–Fahn–Marsden dystonia rating scale (BFMDRS) were used to assess the severity of the patients’ dystonic symptoms and their activities of daily living, respectively. The 36-item Short Form Health Survey (SF-36) [10] was used to evaluate the patients’ health-related quality of life. The SF-36 data of the patients (16 treated with GPi-DBS and 12 with STN-DBS) were available at the 12-month follow-up visit (one GPi patient did not cooperate).

### 2.4. Statistical Analysis

All statistical analyses were performed with SPSS 23.0.0.0 (IBM Corp., Armonk, NY, USA). Descriptive statistics are reported as mean ± standard deviation (SD). Within-group comparisons (i.e., baseline versus 1 month) were performed with a paired *t*-test or Wilcoxon’s signed-rank test. The differences between groups in the clinical results measured after each follow-up were evaluated with a parametric independent-samples *t*-test or the independent-samples Mann–Whitney *U* test. Categorical variables were assessed with Fisher’s exact test. The level of statistical significance was set at 0.05 (two-tailed).

## 3. Results

### 3.1. Demographic and Clinical Characteristics

A total of 29 patients with generalized isolated dystonia underwent DBS, targeting the bilateral GPi in 17 patients and the bilateral STN in 12 patients. The patients’ characteristics and stimulation parameters are shown in Table 1. Nineteen patients (nine GPi and ten STN) were tested for genetic mutations, whereas the others refused genetic testing for personal reasons.

**Figure 1:**
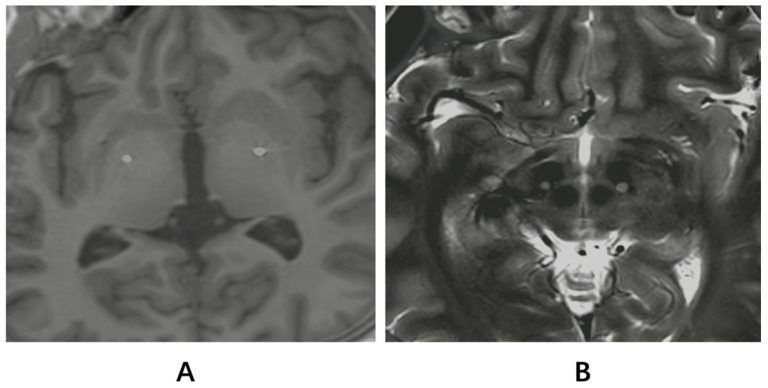
Postoperative electrodeposition. (**A**) The figure shows the fusion of the postoperative CT and preoperative T1-weighted MR images of one person using the Matlab SPM tool. (**B**) The figure shows the fusion of the postoperative CT and preoperative T2-weighted MR images of one person using the Matlab SPM tool. (**A**): GPi. (**B**): STN.

The clinical characteristics of the two groups of patients were similar at baseline in terms of age at surgery (26.12 ± 13.3 years in the GPi group vs. 19.67 ± 16.67 years in the STN group, *p* = 0.116); male sex (58.82% vs. 66.67%, respectively; *p* = 0.717); TOR1A mutation (17.65% vs. 8.33%, respectively; *p* = 0.622); age at onset (17.12 ± 16.75 years vs. 14.08 ± 17.89 years, respectively; *p* = 0.739) and symptom duration (107.18 ± 99.22 months vs. 65 ± 87.18 months, respectively; *p* = 0.422). The age at onset and symptom duration did not influence the outcomes. No significant differences were observed in motor function (BFMDRS-M or BFMDRS-D scores) or quality of life (SF-36) between the two groups at baseline (Table 2). The accuracy of electrode placement was confirmed by the fusion of the postoperative CT and preoperative MR images of one person using Matlab R2023a SPM 12 tool (Figure 1).

### 3.2. Motor Function

**Figure 2:**
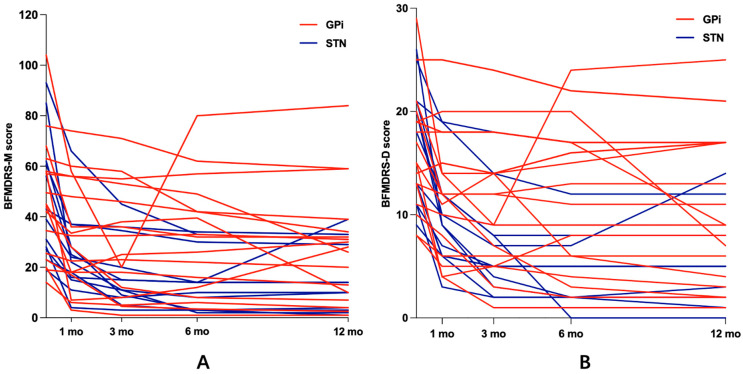
BFMDRS-M scores (**A**) and BFMDRS-D scores (**B**) before surgery and 1, 3, 6, and 12 months after surgery.

The effects of surgery on the total BFMDRS-M and BFMDRS-D scores of the GPi and STN groups are shown in Figure 2. At 1 month after surgery, the percentage improvements were 28.8% and 45.58% for the GPi and STN groups in the total BFMDRS-M score. Both rapid action of stimulation and the microlesion effect are the reason for the decline in clinical improvement in both groups 1 month after surgery. At 3 months after surgery, the percentage improvement in the total BFMDRS-M score was 37.32% in the GPi group and 61.8% in the STN group. At the 1- and 3-month follow-ups, the total BFMDRS-M scores of the two treatment groups declined continuously compared with the preoperative scores, but they did not differ significantly between the groups. After 6 months, the percentage improvement in the BFMDRS-M score relative to baseline was clearly higher in the STN group (63.91%) than in the GPi group (38.36%) (*p* = 0.008). After 12 months, no significant difference was observed in the percentage improvement in the total BFMDRS-M scores of the two groups. Both the STN and GPi groups showed significant improvement in the sub-items of the scale, including for the face (eye, mouth), speech and swallowing, axis (neck, trunk), and limbs (arms, legs). The STN-targeted DBS produced greater percentage improvement on the axis (69.56%) and limbs (65.58%) than the GPi-targeted DBS (40.93% and 36.39%, respectively) at the 6-month follow-up. At the 3-, 6-, and 12-month follow-ups, the percentage improvement in arm symptoms differed significantly after DBS of the STN (70.64%, 80.66%, and 76.89%, respectively) and DBS of the GPi (36.75%, 34.21%, and 38.47%, respectively). No significant intergroup differences were observed in the percentage improvement in the face or speech after surgery (Figure 2A, Table 3).

We recorded significant intergroup differences in the percentage improvement in the total BFMDRS-D scores at 3 months and 6 months but not at 1 month or 12 months. The BFMDRS-D writing score decreased continuously at 1 and 3 months after surgery in the STN group. The percentage improvement in the BFMDRS-D writing score at 1, 3, 6, and 12 months differed significantly between the STN group and the GPi group (Figure 2B, Table 4).

### 3.3. Health-Related Quality of Life

**Figure 3:**
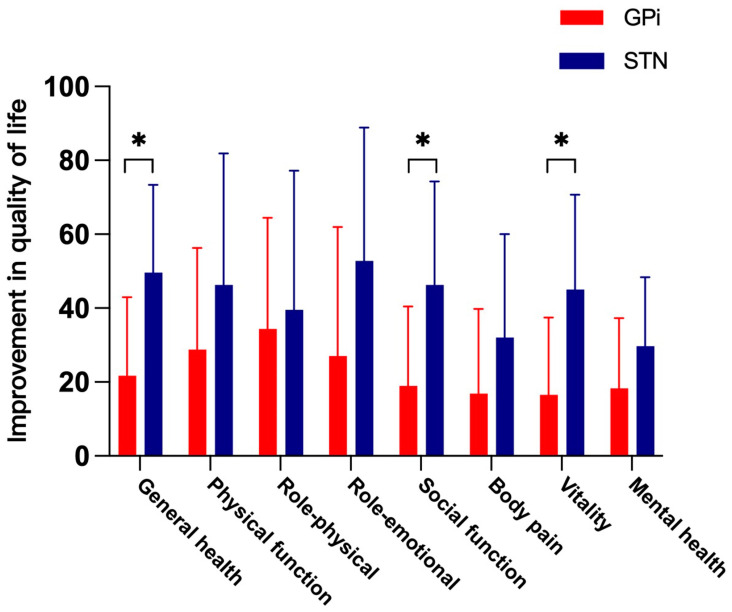
Scores for patient-reported quality of life on the Medical Outcomes Study 36-Item Short-Form General Health Survey (SF-36), with higher scores indicating better daily function and condition. * *p* < 0.05.

At 12 months after surgery, the patients’ quality of life had improved on all subscales of the SF-36 in both groups (Table 5). There were significant differences between the groups in the improvements in general health, social function, and vitality (Figure 3).

### 3.4. Adverse Events

There was no intracranial hemorrhage or infection after DBS surgery in the GPi or STN group. One GPi patient experienced spasm, which was alleviated by lowering the stimulation parameters. One STN patient had a wire fracture at 7 months after surgery, and the wire was replaced in a second operation. In one GPi patient, stimulation was turned off accidentally 4 months after surgery, but was turned on again. We reimplanted a new lead in another operation because one of the leads in the DBS had been misplaced in one STN patient. Dysarthria, vertigo, and paresthesia were experienced by some patients, which were resolved by reprogramming. No dyskinesia was observed in any patient.

## 4. Discussion

Since 1977, DBS has increasingly been recognized as a safe and effective treatment option for patients with dystonia [11]. Several reports have validated the efficacy of GPi-DBS in the treatment of isolated dystonia [12,13,14]. However, as an alternative target of stimulation, Lin et al. considered STN to be a better target option for dystonia in a retrospective trial [7]. However, there have been few reports of the treatment of generalized isolated dystonia with DBS targeting GPi or STN. The present study provides the first evidence that both targets are effective in the treatment of generalized isolated dystonia and improved the health-related quality of life of patients at one institution.

In our previous studies, we suggested that short-term GPi-DBS or short-term STN-DBS significantly affected the motor symptoms of dystonia [15] and demonstrated a significant positive correlation between the percentage improvement in BFMDRS movement scores after short-term DBS and long-term follow-up [15]. The improvement in BFMDRS-M at the 12-month follow-up in the present study is consistent with our earlier research results [15]. The patients with generalized isolated dystonia showed sustained improvement in the BFMDRS-M scores after both GPi- and STN-targeted DBS at 1–3 months after surgery, and a plateau was reached 6 months later. Nonetheless, clinical improvement at 6 months was significantly greater in the STN group compared with the GPi group. The rapid effects of STN stimulation we observed align with findings from earlier studies [7,16], and they may be attributable to the fact that the STN is located in the hyperdirect pathway involved in the basal ganglia–cerebello–thalamo–cortical circuit, as emphasized by Marsden et al. [17]. The STN is known to exert a strong excitatory effect on the output structures of the basal ganglia, including the GPi and substantia nigra pars reticulata. STN-DBS may promote a stronger output from the basal ganglia than GPi-DBS [18]. Patient number 6 showed rapid clinical improvement in her BFMDRS-M scores at 1–3 months after surgery, but her overall clinical symptoms deteriorated unexpectedly at 6 and 12 months. This was caused by the unplanned shutdown of the pulse generator in the 4th month of operation, which could not be recovered by repeated reprogramming. The BFMDRS-M scores of patient number 27 continued to decline at the 1-, 3-, and 6-month follow-ups, but increased suddenly in the 12th month. This may be explained by a wire that fractured at 7 months after surgery and was replaced in a second operation.

The study of Lin et al. proposed that targeting the GPi with DBS has more advantages in terms of axial symptoms than targeting the STN [7]. However, our results are inconsistent with this conclusion. Our results showed that STN stimulation better improved the axis symptoms than GPi stimulation at 6 months after surgery. However, during the 12-month follow-up, no significant difference was detected in the percentage improvement in axial symptoms between the groups. Yin et al. [19] showed that the effects of STN-DBS on isolated cervical dystonia seemed significantly better than those of GPi-DBS. The axial symptoms score on the BFMDRS-M is the sum of the scores for the neck and trunk. The advantages and disadvantages of both targets in addressing axial symptoms require investigation with longer follow-up periods.

After surgery, improvements in individual dystonic symptoms, including those of the face, speech and swallowing, axis, and limbs, were observed in both treatment groups. Moreover, targeting the two regions had similar effects on individual dystonic symptoms during the 12-month follow-up. Based on our results, we have noted that there is no significant variance in effectiveness between the two nuclei for addressing cranial symptoms. However, compared with GPi-DBS, STN-DBS showed greater improvement in arm symptoms at the 3-, 6-, and 12-month follow-ups. Therefore, STN-DBS may be preferable in treating generalized isolated dystonia patients with obvious arm symptoms.

Some studies have reported a significant reduction in the quality of life of patients with focal or generalized dystonia [20]. The SF-36 is the scale most commonly used to measure quality of life, and it includes physical and mental scores for dystonic patients [20,21]. The quality of life of patients with dystonia is improved by DBS targeting either STN or GPi [20,22,23]. However, few studies have investigated the effects of GPi and STN stimulation on the quality of life of patients with isolated dystonia [7]. In this study, stimulation of the STN or GPi improved the eight subscales of the SF-36. General health, social function, and vitality were significantly better after STN-DBS than after GPi-DBS at the 12-month follow-up. The occurrence of adverse events in this study was relatively low, with most being mild and not requiring specific interventions. The predominant adverse effects were associated with stimulation, requiring careful programming to address complications such as dysarthria, vertigo, and paresthesia.

This study had several limitations. First, it was a retrospective study, rather than a randomized controlled trial, of one institution’s experience with 29 cases of generalized isolated dystonia. Therefore, randomized controlled trials are urgently required in the future. Second, the average follow-up time was 1 year, so it is important to extend the follow-up time to determine the longer-term responses to the stimulation of these two targets.

## 5. Conclusions

In conclusion, in this study, we have demonstrated that DBS targeting both the GPi and STN is effective and safe for the treatment of generalized isolated dystonia. However, the choice of the optimal stimulation target should be based on the patient’s symptomatic characteristics. STN-DBS may have more advantages in patients with obvious arm dystonia. STN-DBS had a better clinical effect than GPi-DBS within the first 6 months after surgery.

## Figures and Tables

**Table 1:** Clinical characteristics and treatment parameters with generalized isolated dystonia.

Case	Targets	Sex	Age at Onset (yrs)	Age at Surgery (yrs)	Duration of Symptoms (mos)	Gene Mutation	Stimulation Parameters with Best Response (Contact, Pulse Width, Frequency, Amplitude, Left/Right)
1	GPi	F	1	22	252	NA	Case(+)5(−)/Case(+)2(−), 60/60, 170/170, 2.8/3.2
2	GPi	M	1	25	288	No	Case(+)6(−)/Case(+)2(−), 60/60, 140/140, 3.2/3.5
3	GPi	M	27	29	16	NA	Case(+)5(−)6(−)/Case(+)2(−)3(−), 60/70, 160/160, 3.5/3.0
4	GPi	F	40	45	60	NA	Case(+)6(−)/Case(+)1(−), 60/60, 130/130, 3.4/3.0
5	GPi	M	37	40	36	No	Case(+)6(−)/Case(+)2(−), 60/60, 150/150, 3.5/3.2
6	GPi	F	4	9	60	No	Case(+)6(−)7(−)/Case(+)1(−)2(−), 90/70, 160/160, 2.9/2.8
7	GPi	M	5	29	288	NA	Case(+)7(−)/Case(+)3(−), 60/60, 130/130, 3.1/2.8
8	GPi	M	8	12	48	NA	Case(+)6(−)7(−)/Case(+)1(−)2(−), 100/70, 145/145, 2.95/2.85
9	GPi	M	9	10	6	TOR1A	Case(+)5(−)/Case(+)1(−), 70/80, 150/150, 2.0/2.75
10	GPi	F	36	38	24	NA	Case(+)5(−)6(−)/Case(+)1(−)2(−), 70/80, 140/140, 2.7/2.9
11	GPi	M	6	14	96	TOR1A	Case(+)5(−)/Case(+)1(−)3(−), 80/110, 145/145, 2.65/3.55
12	GPi	M	8	12	48	No	Case(+)6(−)7(−)8(−)/Case(+)2(−)4(−), 60/100, 130/130, 3.5/3.2
13	GPi	M	7	24	204	GNAL	Case(+)5(−)/Case(+)2(−), 90/90, 170/170, 3.4/3.2
14	GPi	M	8	21	156	TOR1A	Case(+)5(−)6(−)/Case(+)1(−)2(−), 80/80, 155/155, 2.2/2.7
15	GPi	F	43	45	24	NA	Case(+)5−/Case(+)2(−), 90/70, 140/140, 3.4/3.4
16	GPi	F	47	50	36	NA	Case(+)6(−)/Case(+)2(−), 90/90, 130/130, 3.4/3.2
17	GPi	F	4	19	180	No	Case(+)9(−)/Case(+)1(−), 60/60, 130/130, 2.6/2.7
18	STN	M	8	13	60	KMT2B	Case(+)7(−)/Case(+)2(−), 80/60, 140/140, 2.1/2
19	STN	M	18	19	12	NA	Case(+)10(−)/Case(+)2(−), 60/60, 130/130, 1.9/2
20	STN	M	66	68	24	No	Case(+)6(−)/Case(+)3(−), 80/70, 150/150, 2/2.25
21	STN	F	29	32	36	No	Case(+)5(−)/Case(+)2(−)3(−), 80/80, 130/130, 2.5/2.4
22	STN	F	7	12	60	NA	Case(+)6(−)/Case(+)2(−), 90/80, 145/145, 2.35/2.25
23	STN	M	4	13	108	No	Case(+)6(−)/Case(+)2(−), 60/60, 130/130, 2.2/2.2
24	STN	M	8	10	24	TOR1A	Case(+)5(−)8(−)/Case(+)1(−)4(−), 70/70, 130/130, 1.5/1.45
25	STN	F	5	6	12	ANO3	Case(+)9(−)/Case(+)1(−), 60/60, 110/110, 1.8/1.9
26	STN	M	3	23	240	No	Case(+)10(−)/Case(+)2(−), 60/60, 130/130, 1.9/1.8
27	STN	F	6	15	84	No	Case(+)5(−)6(−)/Case(+)1(−)2(−), 60/70, 130/130, 1.5/1.55
28	STN	M	9	14	60	No	Case(+)8(−)/Case(+)4(−), 70/60, 140/140, 2.4/1.5
29	STN	M	6	11	60	No	Case(+)7(−)/Case(+)3(−), 60/60, 130/130, 1.9/2.1

F, female; M, male; none, no gene mutation; NA, not available (patient refused genetic testing).

**Table 2:** Baseline characteristics of each patient group.

Characteristic	GPi Group	STN Group	*p* Value
**Demographic or clinical**			
Age at surgery (yrs)	26.12 ± 13.3	19.67 ± 16.67	0.116 ^&^
Age at onset (yrs)	17.12 ± 16.75	14.08 ± 17.89	0.739 ^&^
% males	58.82%	66.67%	0.717 ^#^
% TOR1A mutation	17.65%	8.33%	0.622 ^#^
Symptom duration (mos)	107.18 ± 99.22	65 ± 87.18	0.422 ^&^
**Functional status**			
BFMDRS-M (0–120)	47.09 ± 23.68	47.21 ± 24.32	0.989 *
Eye (0–8)	2.60 ± 2.16	2.75 ± 1.77	NA
Mouth (0–8)	3.19 ± 2.72	2.33 ± 0.58	0.916 ^&^
Speech (0–16)	5.00 ± 4.72	5.20 ± 3.56	0.533 ^&^
Neck (0–8)	5.80 ± 2.21	4.64 ± 2.87	0.332 ^&^
Trunk (0–16)	6.00 ± 4.27	8.00 ± 5.38	0.312 ^&^
Arm (0–32)	13.88 ± 7.94	17.36 ± 9.85	0.312 *
Legs (0–32)	16.50 ± 10.05	16.08 ± 9.06	0.834 ^&^
BFMDRS-D (0–30)	16.18 ± 5.74	17.33 ± 5.74	0.597 *
Speech (0–4)	1.80 ± 1.08	2.20 ± 1.10	0.451 ^&^
Writing (0–4)	2.29 ± 1.10	2.73 ± 1.10	0.315 ^&^
Feeding (0–4)	2.44 ± 1.36	3.09 ± 0.94	0.177 ^&^
Eating and swallowing (0–4)	1.56 ± 0.88	1.20 ± 0.45	0.503 ^&^
Hygiene (0–4)	2.71 ± 1.10	3.08 ± 0.90	0.380 ^&^
Dressing (0–4)	2.59 ± 1.23	3.00 ± 1.04	0.367 ^&^
Walking (0–6)	4.31 ± 1.35	4.42 ± 1.31	0.718 ^&^
**Quality of life**			
SF-36 score			
General health (0–100)	35 ± 12.78	27.92 ± 12.70	0.158 *
Physical function (0–100)	30.94 ± 23.40	18.75 ± 21.33	0.175 ^&^
Role, physical (0–100)	20.31 ± 24.53	18.75 ± 11.31	0.678 ^&^
Role, emotional (0–100)	27.08 ± 30.36	22.22 ± 32.83	0.544 ^&^
Social function (0–100)	35.41 ± 26.69	21.28 ± 18.61	0.212 ^&^
Body pain (0–100)	56.69 ± 31.37	50.17 ± 28.53	0.555 ^&^
Vitality (0–100)	45.32 ± 23.63	40 ± 24.77	0.569 ^&^
Mental health (0–100)	39.75 ± 12.94	44 ± 11.44	0.329 ^&^

* *p* value for comparing corresponding items between the GPi and STN groups derived from a two-tailed independent-samples *t*-test. ^&^ *p* value obtained from a two-tailed Mann–Whitney U test. ^#^ *p* value calculated using Fisher’s exact test.

**Table 3:** BFMDRS-M data in relation to bilateral GPi or STN deep-brain stimulation before surgery and at 1, 3, 6, and 12 months post-surgery.

	BFMDRS-M						*p* Value ^a^				*p* Value (Mean Improvement %) ^b^			
	No. of Patients	Pre	1 mo	3 mo	6 mo	12 mo	1 mo vs. Pre	3 mo vs. 1 mo	6 mo vs. 3 mo	12 mo vs. 6 mo	1 mo	3 mo	6 mo	12 mo
Total														
GPi	17	47.09 ± 23.68	34.09 ± 21.64	29.76 ± 21.25	30.88 ± 22.52	28.21 ± 22.72	** 0.000 ^¥^**	** 0.018 ^¥^**	0.153 ^¥^	0.221 ^¥^	(28.8%)	(37.32%)	** (38.36%) **	(45.73%)
STN	12	47.21 ± 24.32	25.25 ± 16.11	17.71 ± 13.55	14 ± 11.97	15.83 ± 13.57	** 0.001 ^#^**	** 0.002 ^¥^**	** 0.008 ^#^**	1 ^¥^	0.069 ^&^ (45.58%)	0.057 ^&^ (61.8%)	** 0.008 ^&^ (63.91%) **	0.060 * (66.37%)
Eye														
GPi	5	2.6 ± 2.16	1.8 ± 1.68	1.9 ± 1.67	2 ± 2.55	1.4 ± 2.61	0.099 ^#^	0.317 ^¥^	0.854 ^#^	0.317 ^¥^	(34.7%)	(30.67%)	(52%)	(72%)
STN	2	2.75 ± 1.77	0.25 ± 0.35	0.25 ± 0.35	0 ± 0	0 ± 0	NA	NA	NA	NA	NA	NA	NA	NA
Mouth														
GPi	8	3.19 ± 2.72	2.13 ± 2.10	1.75 ± 2.05	1.88 ± 2.3	2 ± 2.2	** 0.039 ^¥^**	0.180 ^¥^	0.655 ^¥^	0.317 ^¥^	(39.58%)	(55.21%)	(64.58%)	(52.08%)
STN	3	2.33 ± 0.58	2 ± 1	1.33 ± 1.53	1 ± 1.73	1 ± 1.73	0.317 ^¥^	0.157 ^¥^	0.317 ^¥^	NA	0.411 ^&^ (16.67%)	0.869 * (50%)	0.911 ^&^ (66.67%)	0.665 ^&^
Face (eyes and mouth)														
GPi	9	4.28 ± 2.53	2.89 ± 2.07	2.61 ± 1.96	2.78 ± 2.39	2.56 ± 2.46	** 0.013 ^¥^**	0.285 ^¥^	0.681 ^#^	0.655 ^¥^	(38.70%)	(42.05%)	(49.23%)	(52.93%)
STN	4	3.13 ± 2.02	1.63 ± 1.11	1.13 ± 1.31	0.75 ± 1.5	0.75 ± 1.5	0.297 ^#^	0.157 ^¥^	0.215 ^#^	NA	0.962 * (37.50%)	0.634 * (54.17%)	0.369 ^&^ (75%)	0.416 ^&^
Speech and swallowing														
GPi	15	5 ± 4.72	4.2 ± 4	4.07 ± 3.83	3.93 ± 3.58	4.07 ± 4.08	0.167 ^¥^	0.854 ^¥^	1 ^¥^	0.785 ^¥^	(20%)	(18.33%)	(21.11%)	(29.44%)
STN	5	5.2 ± 3.56	2.6 ± 1.34	2.20 ± 1.1	2.00 ± 1.22	2.00 ± 1.22	0.144 ^#^	0.317 ^¥^	0.317 ^¥^	NA	0.415 ^&^ (36.67%)	0.348 ^&^ (41.11%)	0.454 ^&^ (43.33%)	0.786 ^&^
Neck														
GPi	15	5.80 ± 2.21	4.87 ± 2.9	3.63 ± 2.91	3.5 ± 2.76	2.9 ± 2.61	0.066 ^¥^	** 0.016 ^¥^**	0.715 ^¥^	0.131 ^¥^	** (20%) **	(44.44%)	** (45.28%) **	(53.06%)
STN	11	4.64 ± 2.87	2.27 ± 1.74	1.73 ± 1.62	0.73 ± 0.79	0.91 ± 0.83	** 0.002 ^#^**	0.063 ^¥^	** 0.026 ^¥^**	0.317 ^¥^	** 0.013 ^&^ (50%) **	0.177 ^&^ (65.15%)	** 0.028 ^&^ (82.58%) **	0.215 ^&^ (73.48%)
Trunk														
GPi	17	6 ± 4.27	4.35 ± 4.24	3.77 ± 4.28	3.9 ± 3.76	2.65 ± 3.32	** 0.003 ^¥^**	0.228 ^¥^	0.892 ^¥^	** 0.039 ^¥^**	(29.74%)	(36.60%)	(39.05%)	(54.74%)
STN	12	8 ± 5.38	4.08 ± 2.39	2.25 ± 1.66	1.92 ± 1.68	2.08 ± 2.31	** 0.018 ^#^**	** 0.002 ^#^**	0.180 ^¥^	0.713 ^¥^	0.712 ^&^ (33.33%)	0.087 ^&^ (60.42%)	0.056 ^&^ (65.28%)	0.713 ^¥^ (69.10%)
Axial (neck and trunk)														
GPi	17	11.12 ± 6.08	8.65 ± 6.47	6.97 ± 6.17	6.97 ± 5.52	5.21 ± 5.04	** 0.003 ^¥^**	0.039 ^¥^	0.438 ^¥^	0.075 ^¥^	(25.04%)	(38.78%)	** (40.93%) **	(52.29%)
STN	12	12.25 ± 7.4	6.17 ± 3.66	3.83 ± 2.92	2.58 ± 2.02	2.92 ± 2.78	** 0.002 ^#^**	** 0.001 ^#^**	** 0.016 ^¥^**	0.713 ^¥^	0.061 ^&^ (40.86%)	0.105 ^&^ (60.15%)	** 0.039 ^&^ (69.56%) **	0.296 ^&^ (68.08%)
Arms														
GPi	17	13.88 ± 7.94	8.85 ± 6.11	7.651 ± 5.92	8.5 ± 7.39	8.24 ± 7.44	** 0.003 ^¥^**	0.072 ^¥^	0.726 ^¥^	0.436 ^¥^	(29.49%)	** (36.75%) **	** (34.21%) **	** (38.47%) **
STN	11	17.36 ± 9.85	8.27 ± 5.55	4.64 ± 2.69	3.27 ± 2.61	4.18 ± 4.07	** 0.002 ^#^**	** 0.011 ^¥^**	** 0.027 ^¥^**	0.414 ^¥^	0.123 ^&^ (45.99%)	** 0.028 ^&^ (70.64%) **	** 0.001 ^&^ (80.66%) **	0.002 * (76.89%)
Legs														
GPi	16	16.5 ± 10.05	12.06 ± 8.65	10.88 ± 8.76	10.75 ± 8.19	10.63 ± 8.94	** 0.018 ^¥^**	0.223 ^¥^	0.473 ^¥^	0.779 ^¥^	** (24.99%) **	(33.88%)	(35.25%)	(40.88%)
STN	12	16.08 ± 9.06	10.08 ± 8.44	8.33 ± 8.44	7.33 ± 7.54	7.83 ± 7.95	** 0.001 ^#^**	** 0.042 ^¥^**	0.089 ^#^	0.593 ^¥^	** 0.101 ^&^ (41.95%) **	0.122 ^&^ (54.57%)	0.065 ^&^ (59.15%)	0.260 * (57.24%)
Limbs (arms and legs)														
GPi	17	29.41 ± 16.64	20.21 ± 14.14	17.88 ± 13.92	18.61 ± 14.61	18.24 ± 15.77	** 0.002 ^¥^**	0.123 ^¥^	0.288 ^¥^	0.770 ^#^	(28.99%)	(36.64%)	** (36.39%) **	(41.75%)
STN	12	32.17 ± 18.35	17.67 ± 13.08	12.58 ± 10.86	10.33 ± 9.55	11.67 ± 11.02	** 0.001 ^#^**	** 0.012 ^¥^**	** 0.015 ^#^**	0.680 ^¥^	0.092 ^&^ (46.08%)	0.073 ^&^ (60.29%)	** 0.024 ^&^ (65.58%) **	0.057 ^&^ (63.38%)

Pre = (preoperative) % improvement from baseline = preoperative BFMDRS score—postoperative BFMDRS score (1, 3, 6, or 12 months)/preoperative BFMDRS score. Values are presented as means ± standard deviations, unless otherwise specified. Bold text denotes statistical significance. ^a^ *p* values indicate the differences in improvement between 1 month and pre-surgery, between 3 months and 1 month, between 6 months and 3 months, and between 12 months and 6 months within each group, analyzed using a two-tailed paired-samples *t*-test (^#^) or a paired-samples Wilcoxon signed-rank test (^¥^). ^b^ *p* values represent the comparison of improvements between GPi and STN at each follow-up, evaluated using a two-tailed independent-samples *t*-test (*) or a Mann–Whitney U test (^&^).

**Table 4:** BFMDRS-D data in relation to bilateral GPi or STN deep-brain stimulation before surgery and at 1, 3, 6, and 12 months post-surgery.

	BFMDRS-D						*p* Value ^a^				*p* Value (Mean Improvement %) ^b^			
	No. of Patients	Pre	1 mo	3 mo	6 mo	12 mo	1 mo vs. Pre	3 mo vs. 1 mo	6 mo vs. 3 mo	12 mo vs. 6 mo	1 mo	3 mo	6 mo	12 mo
Total														
GPi	17	16.18 ± 5.74	12.24 ± 5.89	11.24 ± 6.43	11.41 ± 7.25	10.12 ± 7.19	** 0.002 ^¥^**	0.082 ^¥^	0.670 ^¥^	0.227 ^¥^	(24.46%)	** (30.62%) **	** (30.73%) **	(37.79%)
STN	12	17.33 ± 5.74	10.17 ± 4.88	7.08 ± 4.85	5.92 ± 4.98	6.50 ± 5.52	** 0.000 ^#^**	** 0.000 ^#^**	** 0.041 ^¥^**	0.414 ^¥^	0.051 * (41.62%)	0.009 * (59.73%)	0.005 * (65.56%)	0.05 * (63.08%)
Speech														
GPi	15	1.80 ± 1.08	1.47 ± 0.99	1.47 ± 1.06	1.60 ± 1.18	1.47 ± 1.25	0.059 ^¥^	1 ^¥^	0.458 ^¥^	0.157 ^¥^	(18.89%)	(17.22%)	(13.89%)	(23.89%)
STN	5	2.20 ± 1.10	1.40 ± 1.14	1 ± 1.22	1 ± 1.23	1 ± 1.22	0.099 ^#^	0.157 ^¥^	NA	NA	0.218 ^&^ (40%)	0.151 ^&^ (53.33%)	0.204 ^&^ (53.33%)	0.05 * (53.33%)
Writing														
GPi	17	2.29 ± 1.10	1.76 ± 0.97	1.65 ± 0.93	1.71 ± 0.99	1.71 ± 1.10	** 0.024 ^¥^**	0.480 ^¥^	0.655 ^¥^	1 ^¥^	** (20.10%) **	** (15.20%) **	** (13.73%) **	** (5.88%) **
STN	11	2.73 ± 1.10	1.45 ± 0.82	1 ± 1.22	0.91 ± 0.54	1 ± 0.63	** 0.00 ^¥^**	** 0.025 ^¥^**	0.317 ^¥^	0.317 ^¥^	** 0.03 ^&^ (46.21%) **	** 0.002 ^&^ (66.67%) **	** 0.001 ^&^ (68.94%) **	** 0.003 ^&^ (65.91%) **
Feeding														
GPi	16	2.44 ± 1.36	1.50 ± 1.21	1.38 ± 1.26	1.44 ± 1.46	1.06 ± 1.12	** 0.017 ^¥^**	0.157 ^¥^	1 ^¥^	0.059 ^¥^	(31.25%)	(39.06%)	(36.46%)	(47.92%)
STN	11	3.09 ± 0.94	1.45 ± 0.93	1 ± 0.89	0.73 ± 0.90	0.82 ± 0.98	** 0.003 ^¥^**	0.059 ^¥^	0.18 ^¥^	0.317 ^¥^	0.095 ^&^ (56.06%)	0.114 ^&^ (67.42%)	** 0.048 ^&^ (74.24%) **	0.174 ^&^ (71.97%)
Eating and swallowing														
GPi	9	1.56 ± 0.88	1.00 ± 1.00	1.11 ± 0.93	1.22 ± 0.97	1.22 ± 0.97	0.059 ^¥^	0.317 ^¥^	0.655 ^¥^	NA	(41%)	(29.63%)	(25.93%)	(25.93%)
STN	5	1.20 ± 0.45	0.80 ± 0.44	0.80 ± 0.45	0.60 ± 0.55	0.80 ± 0.45	0.157 ^¥^	NA	0.317 ^¥^	0.317 ^¥^	0.708 ^&^ (30%)	0.938 ^&^ (30%)	0.693 ^&^ (40%)	0.815 ^&^ (30%)
Hygiene														
GPi	17	2.71 ± 1.10	2.06 ± 1.14	1.82 ± 1.29	1.59 ± 1.33	1.29 ± 1.21	** 0.016 ^¥^**	** 0.046 ^¥^**	0.317 ^¥^	0.276 ^¥^	(23.53%)	** (31.37%) **	** (37.75%) **	(45.10%)
STN	12	3.08 ± 0.90	1.92 ± 0.90	1.25 ± 0.97	0.92 ± 0.90	0.92 ± 1.08	** 0.001 ^#^**	** 0.005 ^¥^**	0.180 ^¥^	1 ^¥^	0.175 ^&^ (36.81%)	** 0.033 ^&^ (61.11%) **	** 0.041 ^&^ (70.14%) **	0.1 ^&^ (72.22%)
Dressing														
GPi	17	2.59 ± 1.23	1.88 ± 1.32	1.59 ± 1.23	1.65 ± 1.41	1.35 ± 1.27	** 0.018 ^¥^**	** 0.025 ^¥^**	0.655 ^¥^	0.059 ^¥^	(31.86%)	(39.71%)	** (36.72%) **	(48.53%)
STN	12	3.00 ± 1.04	1.58 ± 1.08	1 ± 1.04	0.67 ± 0.78	0.83 ± 1.03	** 0.003 ^¥^**	** 0.008 ^¥^**	0.102 ^¥^	0.317 ^¥^	0.173 ^&^ (51.39%)	0.074 ^&^ (68.75%)	** 0.031 ^&^ (77.78%) **	0.134 ^&^ (73.61%)
Walking														
GPi	16	4.31 ± 1.35	3.38 ± 1.67	3.13 ± 1.78	3 ± 1.71	2.69 ± 1.58	** 0.007 ^¥^**	0.102 ^¥^	0.680 ^¥^	0.518 ^¥^	(23.75%)	(30.52%)	(32.29%)	(35.63%)
STN	12	4.42 ± 1.31	3.08 ± 1.38	2.33 ± 1.56	2.17 ± 1.70	2.33 ± 1.78	** 0.003 ^#^**	** 0.011 ^¥^**	0.157 ^¥^	0.317 ^¥^	0.567 ^&^ (28.33)	0.087 ^&^ (50.14%)	0.099 ^#^ (53.47%)	0.297 * (50.14%)

Pre = (preoperative) % improvement from baseline = preoperative BFMDRS score – postoperative BFMDRS score (1, 3, 6, or 12 months)/preoperative BFMDRS score. Values are presented as means ± standard deviations, unless otherwise specified. Bold text denotes statistical significance. ^a^ *p* values indicate the differences in improvement between 1 month and pre-surgery, between 3 months and 1 month, between 6 months and 3 months, and between 12 months and 6 months within each group, analyzed using a two-tailed paired-samples *t*-test (^#^) or a paired-samples Wilcoxon signed-rank test (^¥^). ^b^ *p* values represent the comparison of improvements between GPi and STN at each follow-up, evaluated using a two-tailed independent-samples *t*-test (*) or a Mann–Whitney U test (^&^).

**Table 5:** Health-related quality-of-life data for GPi and STN groups before (pre) and 12 months after surgery.

	GPi			STN		
No. of Patients	16			12		
SF-36 Subscale	Pre	12 Month	*p* Value	Pre	12 Month	*p* Value
General health (range, 0–100)	35 ± 12.78	56.69 ± 22.17	**0.001 ^#^**	27.92 ± 12.7	77.5 ± 24.07	**<0.001 ^#^**
Physical function (range, 0–100)	30.94 ± 23.40	59.69 ± 30.96	**0.001 ^#^**	18.7 ± 21.33	65 ± 36.49	**0.002 ^¥^**
Role—physical (range, 0–100)	20.31 ± 24.53	54.69 ± 36.76	**0.003 ^¥^**	18.75 ± 11.31	58.33 ± 37.45	**0.004 ^#^**
Role—emotional (range, 0–100)	27.08 ± 30.36	54.16 ± 36.27	**0.007 ^#^**	22.22 ± 32.83	75 ± 28.88	**<0.001 ^#^**
Social function (range, 0–100)	35.41 ± 26.69	54.33 ± 29.51	**0.003 ^¥^**	21.28 ± 18.61	67.58 ± 30.52	**<0.001 ^#^**
Body pain (range, 0–100)	56.69 ± 31.37	73.56 ± 28	**0.008 ^¥^**	50.17 ± 28.53	82.17 ± 23.33	**0.002 ^#^**
Vitality (range, 0–100)	45.31 ± 23.63	61.88 ± 25.55	**0.002 ^¥^**	40 ± 24.77	85 ± 13.98	**<0.001 ^#^**
Mental health (range, 0–100)	39.75 ± 12.94	58 ± 23.87	**0.002 ^#^**	44 ± 11.44	73.67 ± 21.40	**<0.001 ^#^**

Pre = (preoperative). Values expressed are means ± standard deviations. Scores range from 0 to 100, and a higher score reflects improvement. The *p* values for comparisons of each subscale between 12 months and baseline (preoperative) in each group were assessed using a two-tailed paired-samples *t*-test (^#^) or a paired-samples Wilcoxon signed-rank test (^¥^). Bold text denotes statistical significance.

## Data Availability

Data are contained within the article.
